# Dysregulated Pain–Autonomic Responses in Patients With Nonspecific Chronic Low Back Pain

**DOI:** 10.1155/prm/6637632

**Published:** 2026-07-31

**Authors:** Beatriz Chozas Barrientos, Iara De Schoenmacker, Paulina Simonne Scheuren, Laura Sirucek, David Costa Marques, Robin Lütolf, Lindsay Gorrell, Anke Langenfeld, Mirjam Baechler, Brigitte Wirth, Jan Rosner, Michèle Hubli, Petra Schweinhardt

**Affiliations:** ^1^ Integrative Spinal Research, Department of Chiropractic Medicine, Balgrist University Hospital, University of Zurich, Zurich, Switzerland, balgrist.ch; ^2^ Neuroscience Center Zurich (ZNZ), Zurich, Switzerland; ^3^ Spinal Cord Injury Center, Balgrist University Hospital, University of Zurich, Zurich, Switzerland, balgrist.ch; ^4^ Biomedical Science Lab, Department of Health Sciences and Technology (D-HEST), ETH Zürich, Zürich 8092, Switzerland, ethz.ch; ^5^ Department of Anesthesiology, Pharmacology, and Therapeutics, Faculty of Medicine, University of British Columbia, Vancouver, British Columbia, Canada, ubc.ca; ^6^ International Collaboration on Repair Discoveries (ICORD), Faculty of Medicine, University of British Columbia, Vancouver, British Columbia, Canada, ubc.ca; ^7^ Center for Neuroplasticity and Pain (CNAP), Department of Health Science and Technology, Aalborg University Faculty of Medicine, Aalborg, Denmark; ^8^ Danish Pain Research Center, Department of Clinical Medicine, Aarhus University, Aarhus, Denmark, au.dk; ^9^ Department of Clinical Medicine, Hammel Neurocenter, University Clinic for Neurorehabilitation, Aarhus University, Aarhus, Denmark, au.dk

## Abstract

**Background:**

Neurophysiological assessments might advance the understanding of nonspecific chronic low back pain (nsCLBP) by quantifying altered pain processing and associated autonomic responses. Contact heat‐evoked potentials (CHEPs) and sympathetic skin responses (SSRs) were compared between nsCLBP patients and healthy controls (HCs), and their relationships with experimental and clinical pain ratings were explored.

**Methods:**

A total of 56 nsCLBP patients and 40 age‐ and sex‐matched HCs were included in this study. All participants underwent simultaneous recordings of CHEPs and SSRs in response to two blocks of 15 heat stimuli applied at the back (i.e., the patients’ clinical pain area) and the hand (nonpainful control area). Participants rated the perceived pain intensity of each contact heat stimulus using a numerical rating scale (0–10). The change of pain ratings, CHEP, and SSR amplitudes between blocks was calculated as habituation index.

**Results:**

SSR latencies were prolonged in patients compared to HCs (*F* = 9.5, *p* = 0.003) and were shorter after back stimulation compared to hand stimulation (*F* = 9.6, *p* < 0.001). No other group differences were observed. Clinical pain intensities in patients correlated (1) with contact heat pain ratings in both areas (back: rho = 0.27, *p* = 0.041, control area: rho = 0.272, *p* = 0.042) and (2) inversely with CHEP P2 latencies after hand stimulation (current pain: rho = −0.324, *p* = 0.019; average 4 weeks: rho = −0.320, *p* = 0.022).

**Conclusion:**

Prolonged SSR latencies in nsCLBP might indicate a dysregulated pain–autonomic response.

**Trial Registration:** Clinicaltrials.gov_identifier: NCT04433299

## 1. Introduction

Chronic low back pain (CLBP) is a complex condition, with 80%–95% of primary care cases classified as “nonspecific CLBP” (nsCLBP), because no clear pathoanatomical cause can be identified [1]. Altered pain processing and sensitization of the nociceptive system, which are thought to contribute to nsCLBP [2], can be investigated using neurophysiological techniques [3]. The integrity of thermo‐nociceptive pathways and cortical pain processing can be assessed via contact heat‐evoked potentials (CHEPs) [4, 5]. Sympathetic skin responses (SSRs) allow assessing arousal and autonomic changes by quantifying the activity of sympathetic sudomotor efferent C‐fibers [6], in response to novel or salient stimuli, such as noxious input [7]. A multimodal neurophysiological assessment may help to disentangle the underlying pathophysiological mechanisms by evaluating dysfunctions in nociceptive integration, as assessed by CHEPs, and sympathetic control, as assessed by SSRs.

Habituation is the adaptation to continuous or repetitive stimuli, leading to a reduction in behavioral and neurophysiological responses [8]. Reduced habituation to noxious stimuli is considered a proxy of a sensitized nociceptive system [2] and has been shown in different readouts and cohorts, such as fibromyalgia and migraine patients, compared to healthy controls (HCs) [8].

CHEPs, SSRs, and habituation provide distinct yet complementary insights into altered nociceptive processing, as well as autonomic dysregulations, which might contribute to nsCLBP [9]. Linking these readouts to patients’ experimental and clinical pain perception might offer new insights into underlying pathophysiological mechanisms. Yet, to the best of our knowledge, these readouts have not been studied in combination in nsCLBP. Therefore, the aims of this exploratory study were as follows: a) to compare CHEP and SSR readouts between nsCLBP patients and HCs, and b) to correlate CHEP and SSR readouts with participants’ ratings of contact heat pain stimuli and with patients’ clinical pain, in order to investigate the association of cortical and autonomic readouts with pain perception [10].

## 2. Methods

This study is a part of a bigger project (Clinical Research Priority Program [CRPP] “Pain,” https://www.crpp-pain.uzh.ch/en.html), in which a comprehensive test battery evaluating clinical characteristics and measures involving neurophysiological readouts, experimental pain perception, magnetic resonance imaging, and questionnaires was performed in a larger group of participants, including other patient cohorts. For the purpose of this study, only the neurophysiological and questionnaire data of nsCLBP patients and HCs were used.

### 2.1. Participants

Sixty‐four patients with nsCLBP were recruited via Balgrist University Hospital and advertisements in Swiss chiropractic practices or UZH Marktplatz (online platform) between 2019 and 2022. Inclusion criteria for patients were age between 18 and 80 years and nsCLBP [11], lasting for more than 3 months and without signs of specific causes for their back pain (including infection, malignancy, rheumatic and systematic inflammatory disorders, and fracture). Forty‐eight age‐ and sex‐matched HCs were recruited. Exclusion criteria were any major medical or psychiatric condition that affects physical capacity or pain sensitivity (i.e., severe heart disease and diabetes) and pregnancy. Inclusion and exclusion criteria were first checked via phone screening and again in a clinical session (patient history and clinical examination) performed by a clinician (physiotherapist or chiropractor), who additionally assessed participants’ height and weight. Body weight was measured using a standard weighing scale. Height was measured using a measuring tape fixed vertically to a wall. Participants wore a shirt, shorts, and no shoes during measuring procedures.

All participants provided written informed consent before study participation. The study was approved by the local ethics committee Kantonale Ethikkommission Zürich (Nr.: 2019–00136) and performed in accordance with the guidelines of the Declaration of Helsinki.

### 2.2. Questionnaires

Participants electronically completed questionnaires to assess anxiety and depression (Hospital Anxiety and Depression Scale; HADS [12]; score range: 0–21, 0–7 indicates *normal levels of anxiety or depression*, 8–10 *mild levels*, 11–15 *moderate levels*, and 16–21 *severe levels*), which has been validated in several patient groups [13], including acute low back pain [14], and pain catastrophizing (Pain Catastrophizing Scale; PCS [15]; score range: 0–52, ≥ 30 indicates *a clinically significant level of pain catastrophizing*).

Additionally, nsCLBP patients completed questionnaires regarding their clinical pain, including the current pain intensity (“how would you rate your pain right now?” based on [16]) and the average pain intensity in the past four weeks (“on average, how severe has your pain been over the past four weeks?” based on [17]) on a scale from 0 to 10, as well as the self‐reported duration of their low back pain. The current pain intensity reflected the pain status of patients at baseline, right before taking part in the study, while the average pain intensity in the past four weeks was assessed to capture a more generalized pain status, within the recommended time frame for pain recall [18].

### 2.3. Stimulation Procedure

Four blocks of 15 phasic contact heat stimuli were applied to two testing sites (the individual patient’s most painful site of the lower back, between the 12th thoracic and 2nd sacral spinal levels) and the hand dorsum at the first interosseous space), i.e., two blocks per site, in randomized order with a 2‐min break between blocks. The testing site on the back was the most painful area self‐reported by each patient. The same testing site was used for the HC matched individually to each patient. The dorsum of the nondominant hand served as pain‐free control area.

Contact heat stimuli (increased baseline temperature of 42°C, destination temperature of 52°C, and ramp of 70°C/s [19]) were applied with a 13‐ to 17‐s interstimulus interval (ISI), using a 27 mm diameter CHEP thermode (PATHWAY Pain & Sensory Evaluation System, Medoc Ltd., Ramat Yishai, Israel). After reaching the destination temperature, the thermode temperature declined and returned to baseline. The thermode was held manually and slightly moved between stimuli to minimize peripheral adaptation or sensitization. An increased baseline temperature was used because it results in an improved CHEP acquisition with increased N2P2 amplitudes and reduced N2 latency between‐subject variability [20, 21]. When the increased baseline stimulation was not tolerated, a baseline temperature of 35°C (normal baseline temperature) was used. Participants had to rate the perceived pain for each heat stimulus using a numeric rating scale (NRS) from 0 to 10: 0 being *no pain*, 1 *the slightest pain sensation*, and 10 *the most intense pain tolerable* [22].

CHEPs are a validated [23] and widely used method for assessing spinothalamic nociceptive pathway function in healthy subjects and clinical populations, including chronic pain conditions [24, 25]. CHEPs were used in the present study due to their noninvasiveness, safety, and reliability for repeated measurements [20, 26].

### 2.4. Recording Procedure

Time‐locked CHEPs and SSRs were recorded in the prone position during the application of contact heat stimuli. Recording sites were prepared with skin prep sandpaper tape (Red Dot Trace Prep, 3M) and alcohol (Softasept N, B Braun, Germany).

CHEPs were recorded with 9 mm Ag/AgCl cup electrodes filled with conductive adhesive gel (Elefix). Cup electrodes were positioned on the vertex (Cz), considered the most reliable landmark to record N2 and P2 waves [27], and referenced to the earlobes (A1‐A2) according to the 10–20 system [26]. CHEP signals were sampled at 2000 Hz using a preamplifier (20,000x, ALEA Solutions) and bandpass filtered in the range 0.5–30 Hz. Data were recorded within a 1‐s pretrigger and 9‐s post‐trigger window in a customized program based on LabVIEW (V2.6.1., CHEP, ALEA Solutions). CHEP signals contaminated with movement artifacts were excluded online, and additional heat stimuli were applied to obtain an averaged evoked potential of 15 artifact‐free signals per block. Offset correction based on the 500 ms prestimulus window was used on the averaged CHEP before further analyses [28]. CHEP latencies were defined as P2: maximal positive deflection of the signal 200–700 ms post‐trigger and higher than 2 standard deviations (SD) of the noise signal (500 ms pretrigger time window); and N2: maximal negative deflection of the signal before P2, and higher than 2 SD of the noise signal. CHEP amplitudes were defined as peak‐to‐peak (N2P2) responses.

SSRs were recorded with surface electrodes (Ambu BlueSensor NF, Ballerup, Denmark) attached to the dominant hand, with the active electrode attached to the palm, and the reference electrode to the hand dorsum [28]. Heating lamps were used to maintain a constant skin temperature (≥ 32°C) of the recording and stimulation sites because skin temperature has been shown in influence SSR recordings [29]. SSRs were measured as the voltage difference between the active and the reference electrode (µV). SSRs were sampled at 2000 Hz with a preamplifier and a 0.1–12 kHz bandpass filter. Data were recorded within a 1‐s pretrigger and 9‐s post‐trigger window in a customized program based on LabVIEW (V2.6.1., CHEP, ALEA Solutions). Signals contaminated with movement artifacts or non‐time‐locked responses were excluded offline. SSR latencies (i.e., first deflection point of the signal) and amplitudes (i.e., peak‐to‐peak responses) were detected by a customized algorithm in R statistical software. SSR latencies and amplitudes were inspected for each SSR signal to ensure the correct detection by the algorithm [28].

The habituation index (HI) was calculated as ((block2–block1)/block1) x 100, whereby block1 and block2 are the means of pain ratings, CHEP, or SSR amplitudes for the respective block. Negative values represent habituation; positive values represent sensitization.

### 2.5. Statistical Analysis

Statistical analyses were performed using R statistical software (Version 4.1.0. for Windows) and chosen according to the data distribution assessed via inspection of histograms and quantile–quantile plots. Age, height, weight, and questionnaire scores were compared between nsCLBP and HCs with Student’s *t*‐tests. The distribution of men and women was compared between nsCLBP and HCs using a chi‐square test of independence. The statistical significance was set at *p* < 0.05.

Dependent variables were contact heat pain ratings, CHEP, and SSR amplitudes and latencies as well as HI of contact heat pain ratings, CHEP, and SSR amplitudes. Independent variables were cohort (nsCLBP and HCs) and testing site (back and hand).

Separate linear mixed‐effect models were performed to test the effect of cohort and testing site on contact heat pain ratings, CHEP, and SSR parameters, i.e., amplitudes, latencies, and HI (%). Age, height, and stimulation baseline temperature were included as covariates because there is strong evidence that they might have an impact on neurophysiological readouts [26]. Because height differences between females and males seem to largely explain sex‐related differences in neurophysiological findings, sex was purposely not included as a covariate in the models. The interaction “cohort x area” was included in all models, and “participant” was added as a random effect. Planned post hoc comparisons were performed using Student’s *t*‐tests.

To explore the relationships of neurophysiological measures with experimental pain ratings and clinical pain intensities, Spearman’s correlation analyses were performed between CHEP and SSR amplitudes, latencies, HI, and (a) contact heat pain ratings (all participants) and (b) current clinical pain intensity and average pain intensity in the past four weeks (patients only). Additionally, CHEP N2P2 amplitudes and SSR amplitudes were correlated with contact heat pain ratings because amplitudes of neurophysiological readouts have been shown to be related to perceived pain intensity [30–33]. This correlation analysis was performed independently for each testing site, and due to its exploratory nature, Spearman’s rho values and *p*‐values are reported. To assess the robustness of potentially significant correlations, a Bonferroni–Holm correction for multiple comparisons (20 in total for each stimulation area: 9 readouts each correlated with two clinical pain readouts and contact heat pain ratings correlated with CHEP N2P2 and SSR amplitudes) was also performed.

## 3. Results

### 3.1. Cohort Characteristics

Of the recruited 64 nsCLBP patients and 48 HCs, 8 participants were excluded from the study due to discontinuation (3 patients), abnormal sensory findings (2 HCs; one with absent somatosensory evoked potentials, possibly related to a past disk herniation, and one with pathological temperature perception unable to clearly discriminate cool and warm stimuli on bedside testing, possibly related to age above 65 where sensory abnormalities are more common), suspected neurological (1 patient) or psychiatric (1 patient) conditions, or development of low back pain between the inclusion and the visit (1 HC). Ten of the remaining 45 HCs had been matched to another chronic pain cohort of the CRPP “Pain,” and they were only included in analyses if the hand was used as the control area, for a total of 41 HCs. Additionally, 4 participants did not complete the CHEP session because they refused (1 patient) or because the heat stimuli were too painful (2 patients and 1 HC), leaving a total of 56 nsCLBP patients and 40 HCs.

The 56 nsCLBP patients and 40 HCs did not differ regarding age, height, or weight (Table 1), which are factors that can influence neurophysiological readouts [27]. Experimental pain ratings and values of relevant neurophysiological readouts are reported in Table 2.

**TABLE 1:** Baseline characteristics of nsCLBP and HC cohorts.

	HC	nsCLBP	Test statistic	*p*‐value
Number	40	56		
Sex, % female	55.0	62.5	0.488	0.485
Age (years)	48.5 (17.2)	50.7 (16.6)	−0.614	0.541
Height (cm)	171.8 (8.5)	170.3 (7.9)	0.852	0.397
Weight (kg)	70.2 (12.2)	69.9 (12.5)	0.072	0.943
HADS Anxiety	3.5 (2.67)	5.1 (3.41)	−2.593	0.0336^∗^
HADS Depression	1.7 (2.15)	3.9 (3.31)	−3.962	< 0.001^∗^
PCS	5.1 (6.49)	12.9 (9.65)	−4.599	< 0.001^∗^

*Note:* Data are shown as mean (standard deviation, SD). Test‐statistic reflects chi‐square for sex (%) and t‐test for all remaining variables (i.e., age, height, weight, HADS, and PCS).

Abbreviations: HADS, Hospital Anxiety and Depression Scale; HC, healthy controls; nsCLBP, nonspecific chronic low back pain; PCS, Pain Catastrophizing Scale.

^∗^ *p* < 0.05, statistically significant difference.

**TABLE 2:** Baseline (stimulation block 1) pain ratings and neurophysiological parameters.

	HC		nsCLBP	
	Hand	Back	Hand	Back
Experimental pain (NRS)	4.1 (1.9)	5.2 (2.2)	4.0 (2.4)	5.4 (2.5)
N2P2 amplitude (μV)	33.6 (13.7)	33.2 (12.2)	27.9 (15.5)	30.6 (16.5)
N2 latency (ms)	293.9 (39.4)	293.9 (51.6)	302.6 (38.8)	302.0 (49.4)
P2 latency (ms)	428.1 (46.6)	439.1 (66.1)	442.9 (48.8)	442.6 (56.0)
SSR amplitude (μS)	2.79 (2.6)	3.79 (3.1)	2.33 (2.5)	3.99 (3.3)
SSR latency (s)	1.77 (0.2)	1.73 (0.2)	1.90 (0.2)	1.82 (0.2)

*Note:* Data are shown as mean (SD).

Abbreviations: HC, healthy controls; NRS, numerical rating scale; nsCLBP, nonspecific chronic low back pain; SSR, sympathetic skin responses.

nsCLBP patients had a median pain duration of 60 months (range 4–670), a median current pain intensity of 3 (range 0–10), and a median 4‐week average pain of 4 (range 0–8 NRS). nsCLBP patients had higher PCS, anxiety, and depression scores compared to HCs (Table 1). Patients’ most painful area was located between the level of the second lumbar (L2) and second sacral (S2) vertebrae (Table 3). The increased stimulation baseline was used in 87.5% of nsCLBP patients and 91.2% of HCs (chi‐square(1) = 0.344, *p* = 0.558). Because statistical comparison between normal and increased baselines would not have been meaningful due to the low number of participants who underwent the normal baseline protocol (4 HCs and 8 nsCLBP), both stimulation protocols were analyzed together.

**TABLE 3:** Segmental levels of the most painful area of the nsCLBP patients.

	Segmental level	Side	Number of patients (% of total)
Most painful area	L1	Left	0 (0)
		Right	0 (0)
	L2	Left	4 (7.4)
		Right	2 (3.5)
	L3	Left	6 (10.7)
		Right	0 (0)
	L4	Left	7 (12.5)
		Right	7 (12.5)
	L5	Left	8 (14.2)
		Right	9 (16.1)
	S1 or S2	Left	7 (12.5)
		Right	6 (10.7)

*Note:* L1/2/3/4/5: lumbar, S1/2: sacral 1/2.

Abbreviation: nsCLBP, nonspecific chronic low back pain.

### 3.2. Contact Heat Pain Ratings

There was no interaction between cohort and stimulation area (F(1, 91.58) = 1.037, *p* = 0.311) nor did contact heat pain ratings differ between cohorts (F(1, 89.45) = 0.0008, *p* = 0.978). Contact heat pain ratings differed between testing sites, with higher pain ratings when stimulating the back compared to the hand (F(1, 92.81) = 51.410, *p* < 0.001).

There was no interaction between cohort and testing site for the HI of contact heat pain ratings (F(1, 93.82) = 0.149, *p* = 0.700), and HI of contact heat pain ratings was not different between cohorts (F(1, 91.13) = 1.968, *p* = 0.164) nor between testing sites (F(1, 94.22) = 2.487, *p* = 0.118). To test whether habituation did occur between blocks 1 and 2, it was tested whether HI was different from 0 across cohorts and testing sites using a one‐sample *t*‐test. The HI of contact heat pain ratings was −17.45% on average and significantly different from 0 (*t*(190) = −9.972, *p* < 0.001), indicating that there was overall habituation of contact heat pain ratings.

In nsCLBP, contact heat pain ratings correlated positively with current clinical pain for stimulation of the back (rho = 0.274, *p* = 0.041) and the hand (rho = 0.272, *p* = 0.042). These correlations were no longer significant after Bonferroni correction. There was no significant correlation of contact heat pain ratings with the clinical pain in the past 4 weeks (hand: rho = 0.131, *p* = 0.341; back: rho = 0.249, *p* = 0.067).

### 3.3. CHEPs

There was no interaction between cohort and testing site for CHEP N2 latencies (F(1, 74.16) = 0.139, *p* = 0.711), P2 latencies (F(1, 86.96) = 0.877, *p* = 0.352), or N2P2 amplitudes (F(1, 84.86) = 2.096, *p* = 0.151). Latencies and amplitudes were not different between cohorts (N2 latency: F(1, 78.36) = 1.773, *p* = 0.189; P2 latency: F(1, 90.45) = 1.064, *p* = 0.305; N2P2 amplitude: F(1, 87.56) = 2.120, *p* = 0.141) nor between testing sites (N2 latency: F(1, 74.79) = 1.018, *p* = 0.316; P2 latency: F(1, 87.63) = 0.575, *p* = 0.450; N2P2 amplitude: F(1, 86.02) = 0.953, *p* = 0.332). Age and height had an influence on all three readouts (age: F’s > 14.823, *p*‐values < 0.05; height: F’s > 12.946, *p*‐values < 0.05). Baseline temperature had a significant effect on both N2 and P2 latencies (F’s > 22.750, *p*‐values < 0.05) but not on amplitudes.

Similar to HI of contact heat pain ratings, there was no interaction effect between cohort and testing site for HI of N2P2 amplitudes (F(1,168) = 1.329, *p* = 0.251), and HI of N2P2 amplitudes was not different between cohorts (F(1,168) = 0.217, *p* = 0.642) nor between testing sites (F(1,168) = 0.152, *p* = 0.698). The HI of N2P2 amplitudes across cohorts and stimulation areas was −17.46% on average and significantly different from 0 (*t*(174) = −8.441, *p* < 0.001), meaning that there was overall habituation of N2P2 amplitudes.

Patients’ N2P2 amplitudes and the respective HI did not correlate with their current clinical pain nor with the average clinical pain during the past four weeks (rho’s < 0.208, *p*‐values > 0.05). Patients’ CHEP P2 latencies after hand stimulation correlated negatively with their current clinical pain (rho = −0.324, *p* = 0.019) and with their average pain in the past 4 weeks (rho = −0.320, *p* = 0.022). These correlations were no longer significant after Bonferroni correction. Patients’ CHEP P2 latencies after back stimulation, and N2 latencies for both stimulation areas, did not correlate with their current clinical pain nor with the average clinical pain during the past four weeks (rho’s < −0.275, *p*‐values > 0.05).

### 3.4. SSRs

**FIGURE 1:**
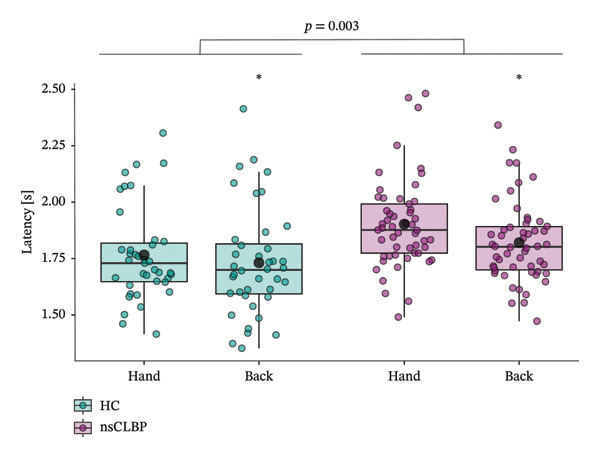
Sympathetic skin response (SSR) latencies for healthy controls (HCs) (blue) and nonspecific chronic low back pain patients (nsCLBP) (purple) for stimulation of the hand and the back. SSR latencies were prolonged in nsCLBP patients compared to HCs, and SSR latencies were shorter when stimulating the back compared to the hand. Depicted are the medians (horizontal solid line) and the means (black point). The whiskers indicate the variability outside the upper and lower quartiles. ^∗^ *p* < 0.05, significant main effect of area.

There was no significant interaction between cohort and stimulation area for SSR latencies (F(1, 90.51) = 1.565, *p* = 0.214). SSR latencies differed between cohorts (F(1, 89.44) = 9.316, *p* = 0.003), with nsCLBP presenting with prolonged SSR latencies compared to HCs. In addition, there was a main effect of testing sites (F(1, 91.55) = 9.650, *p* = 0.003) with faster SSR latencies when stimulating the back (Figure 1).

**FIGURE 2:**
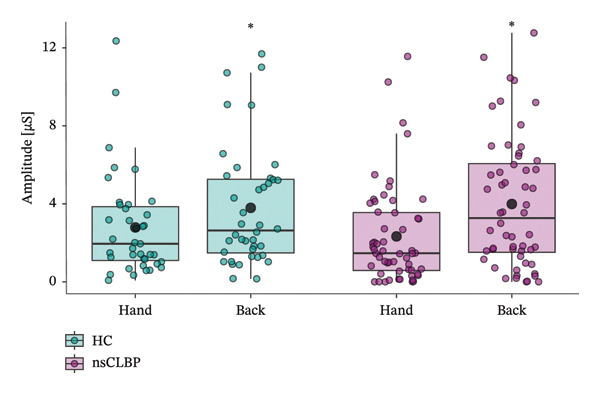
Sympathetic skin response (SSR) amplitudes for healthy controls (HCs) (blue) and nonspecific chronic low back pain patients (nsCLBP) (purple) for stimulation of the hand and the back. SSR amplitudes were increased when stimulating the back compared to the hand. Depicted are the medians (horizontal solid line) and the means (black point). The whiskers indicate the variability outside the upper and lower quartiles. ^∗^ *p* < 0.05, significant main effect of area.

There was no significant interaction between cohort and stimulation area (F(1, 92.13) = 1.868, *p* = 0.175) for SSR amplitudes, and SSR amplitudes were not different between cohorts (F(1, 90.76 = = 0.0001, *p* = 0.990). However, SSR amplitudes were different between testing sites (F(1, 93.37) = 39.316, *p* < 0.001), with higher amplitudes when stimulating the back compared to the hand (Figure 2). There was a significant effect of age on SSR amplitudes (F(1, 90.43) = 5.810, *p* = 0.018), with older participants presenting with decreased amplitudes (*t* = −2.41).

There was no significant interaction between cohort and stimulation area (F(1, 84.21) = 1.500, *p* = 0.224) for HI of SSR amplitudes, and HI of SSR amplitudes was not different between cohorts (F(1, 84.41) = 0.020, *p* = 0.888), nor between testing sites (F(1, 84.99) = 0.004, *p* = 0.951). The HI of SSR amplitudes across cohorts and stimulation areas was −48.54% and significantly different from 0 (*t*(163) = −17.831, *p* < 0.001), meaning that there was overall habituation of SSR amplitudes.

SSR amplitudes and contact heat pain ratings did not significantly correlate in HCs nor in nsCLBP patients (*p*‐values > 0.05). Patients’ SSR amplitudes, latencies, and HI did not correlate with their clinical pain readouts (*p*‐values > 0.05).

## 4. Discussion

In this study, patients with nsCLBP showed prolonged contact heat‐induced SSR latencies compared to HCs. These results suggest a dysregulated pain–autonomic response in nsCLBP patients.

### 4.1. Pain‐Autonomic Interaction: SSRs

Patients with nsCLBP showed longer SSR latencies than HCs, in line with existing literature on other pain patient cohorts: fibromyalgia [6], chronic prostatitis [34], complex regional pain syndrome [35], chronic neuropathic pain after surgery [36], and CLBP patients with or without failed back surgery syndrome [37]. The prolonged latencies in nsCLBP patients were observed in the present study both for stimulation at their most painful area in the back and at a pain‐free remote body area. This, coupled with the normal afferent system response reflected by unaltered CHEPs, might indicate a disruption in the efferent system’s response to painful stimuli. Such disruption might be associated with a generalized higher alertness and arousal to nociceptive input in nsCLBP patients. This notion is supported by other measures of autonomic dysfunction, such as increased resting heart rate and decreased heart rate variability, which are consistently altered in several chronic pain cohorts, including CLBP [38].

The sympathetic nervous system plays a key role in regulating the response to external stimuli or threats, including pain [39]. Therefore, an imbalance of the autonomic function, driven, for example, by increased sympathetic drive, could be a risk factor for developing pain or an obstacle for adaptively coping with pain [38]. The changes observed in different autonomic nervous system readouts in the previous studies discussed in the above paragraph, together with the present results of prolonged SSR latencies, might point toward a generalized dysregulated pain–autonomic interaction in nsCLBP patients. Future studies investigating these responses in relation to both painful and nonpainful stimuli would help to discern pain‐related autonomic alterations from general autonomic nervous system alterations.

SSR latencies were shorter in response to back compared to hand stimulation in nsCLBP patients as well as HCs. This might be explained by the shorter afferent pathway for the back compared to the hand. An alternative explanation could be that the back is more sensitive to warming than other body regions, such as the arms or the hands [40, 41], which might be associated with faster central processing. The observation that contact heat pain ratings and SSR amplitudes were higher after back stimulation compared to hand stimulation supports this interpretation [9, 42]. Future research is needed to better understand whether the lumbar region of nsCLBP patients is indeed more sensitive to warming than other distal, nonaffected body areas.

In contrast to latencies, SSR amplitudes did not differ between nsCLBP patients and HCs. Further, SSR amplitudes did not correlate with contact heat pain ratings in either cohort, nor with clinical pain readouts in nsCLBP. This illustrates that an increased autonomic response does not necessarily relate to increased pain perception and suggests that autonomic responses are partially independent of perceptual processes. Autonomic responses may depend more on direct spinal somatosympathetic reflexes and brainstem mechanisms involved in both nociceptive and autonomic function [39]. Nevertheless, some studies report an association between autonomic responses and pain perception [10, 28]. Thus, it remains to be determined under which circumstances there is a close correspondence between autonomic responses and the perceived pain intensity.

### 4.2. Cortical Pain Processing: CHEPs

None of the CHEP parameters differed between cohorts. In a publication using data from the same study, in which the current nsCLBP cohort was grouped together with complex regional pain syndrome and neuropathic pain patients as one chronic pain cohort [43], CHEP latencies were prolonged compared to HCs. As shown in the present study, this difference was not driven by nsCLBP patients. This aligns with previous work [44] that found no differences in laser evoked potentials (LEPs), a technique similar to CHEPs, between nsCLBP patients and HCs, neither for the back nor the abdomen. In fact, studies reporting differences in CHEP readouts between nsCLBP patients and HCs generally tested cohorts with specific back pain causes, such as persistent spinal pain syndrome after surgery [42] or radiculopathy [45]. In the current study, nsCLBP patients’ contact heat pain ratings after back and hand stimulation positively correlated with their current clinical pain but not with the pain in the last four weeks, suggesting that experimental pain sensitivity might be more dependent on the current state of the patient than a more generalized pain state. This association, albeit no longer significant when correcting for multiple comparisons, might be mediated by psychological states, such as pain catastrophizing or pain‐related fear [46]. CHEP N2P2 amplitudes and contact heat pain ratings were not correlated in either cohort. This finding aligns with another study in HCs, where no correlation between N2P2 amplitudes and pain ratings was observed using LEPs [47]. However, a body of literature has reported a positive association between amplitudes, pain ratings [30–33], and stimulus intensity [48] in HCs. Thus, the details on the relationship between CHEP/LEP amplitudes and pain perception and the influence of factors, such as saliency or stimulus pain intensity [33, 49], need to be better understood.

CHEP P2 latencies after hand stimulation were negatively correlated with patients’ clinical pain readouts, although not significantly after Bonferroni correction. The P2 component is considered to be involved in the subjective experience of pain [50]. Therefore, faster latencies in patients with higher clinical pain might be indicative of facilitated cortical processing, potentially linked to higher alertness during higher pain. A study on fibromyalgia patients found prolonged LEP P2 latencies compared to HCs [6], while a study on CLBP patients [51] reported a reduced P260 (a somatosensory‐specific evoked potential correlated with the multimodal P2) amplitude compared to HCs for electrical stimulation of the back and of the abdomen. The contradicting results of these studies might be impacted by the different protocols and stimulus modalities used. Furthermore, they did not report on correlations between P2 and clinical pain. Literature on P2 latencies and pain intensities is scarce; thus, more work is needed to better understand the nature of this relationship. No other significant correlations were found between the remaining CHEP parameters (CHEP N2 latency, N2P2 amplitudes, and N2P2 HI) and clinical pain readouts in nsCLBP patients.

### 4.3. Habituation

Habituation parameters were not different in nsCLBP compared to HCs, nor between testing sites. These negative findings align with studies in fibromyalgia [6] and spinal cord injury patients [36]. A systematic review [8] reported that most studies comparing CLBP patients and HCs showed no cohort differences in habituation of pain ratings and brain activity. However, in patients with painful radiculopathy [52], habituation of LEP N2P2 amplitudes was decreased compared to HCs, and those patients with signs of central sensitization (dynamic mechanical allodynia and hyperalgesia at the affected dermatome) presented more impaired LEP habituation compared to patients without such signs. In another study in CLBP, reduced habituation in response to noxious and non‐noxious stimuli was observed using event‐related electroencephalogram recordings [53]. The absence of reduced habituation in the present nsCLBP cohort might indicate that central sensitization is not a primary driver in these individuals, even though signs of spinal sensitization were observed in a similar cohort from the CRPP “Pain” [54]. These conflicting habituation findings might be due to differing relative contributions of pain mechanisms in this heterogeneous group of nsCLBP patients.

## 5. Limitations

The main limitations of this study are related to its methodological and sample characteristics, which constrain the interpretation of the findings. Firstly, contact heat stimuli applied in this study were exclusively noxious. As a result, the current findings do not make it possible to disentangle autonomic alterations specifically related to pain and those that would reflect more general autonomic nervous system activity. Future research on autonomic alterations should incorporate both painful and nonpainful stimuli to better differentiate between responses associated with different types of sensory experiences. Secondly, contact heat stimulation is an often used and reliable method to elicit robust experimental pain. Using brief stimuli and steep ramps is particularly suited for studying neurophysiological and autonomic responses. However, because such stimuli differ substantially from the more gradual and diffuse pain typically experienced in CLBP, the ecological validity of this model might be limited. Thirdly, nsCLBP is a highly heterogeneous condition, with patients differing in the relative contributions of underlying pain mechanisms. This heterogeneity limits the interpretability of our findings. Future studies should incorporate more precise stratification (e.g., based on nociceptive, neuropathic, and nociplastic pain categories) to improve result interpretation.

## 6. Conclusion

In summary, the findings suggest a dysregulated pain–autonomic response in nsCLBP patients. Neurophysiological methods, such as SSRs, have the potential to track and shed light on pain‐autonomic processing and to better understand differences between individuals with nsCLBP. This dysregulation seems to arise from the efferent system, possibly linked to hypervigilance/arousal. These findings suggest that targeting the regulation of the efferent autonomic nervous system may represent a promising avenue for future clinical pain management strategies and research.

## Author Contributions

This study was designed by Petra Schweinhardt, Michèle Hubli, Laura Sirucek, Iara De Schoenmacker, Robin Lütolf, Paulina Simonne Scheuren, and Jan Rosner. The experiments were performed by Laura Sirucek, Iara De Schoenmacker, Robin Lütolf, Paulina Simonne Scheuren, and Lindsay Gorrell. Patients were examined by Lindsay Gorrell, Anke Langenfeld, Brigitte Wirth, and Mirjam Baechler. The data were analyzed by Beatriz Chozas Barrientos, Iara De Schoenmacker, Paulina Simonne Scheuren, and David Costa Marques, and the results were critically examined by all authors. Beatriz Chozas Barrientos had a primary role in preparing the manuscript, which was mainly edited by Laura Sirucek, Iara De Schoenmacker, Michèle Hubli, and Petra Schweinhardt.

## Funding

The study was funded by the Clinical Research Priority Program, the Theodor und Ida Herzog‐Egli Stiftung, and the Lundbeck Foundation. Open access publishing facilitated by Universitat Zurich, as part of the Wiley ‐ Universitat Zurich agreement via the Consortium of Swiss Academic Libraries.

## Disclosure

All authors have approved the final version of the manuscript and agreed to be accountable for all aspects of the work.

## Conflicts of Interest

The authors declare no conflicts of interest.

## Data Availability

The data that support the findings of this study are available on request from the corresponding author. The data are not publicly available due to privacy or ethical restrictions.
